# Supplementary material for: Binocular combination in the autonomic nervous system
Source: PLoS One. 2026 Sep 1;21(9):e0356765. doi: 10.1371/journal.pone.0356765 (PMC13533428; doi:10.1371/journal.pone.0356765)
Supplement: Supplementary file 1 [file pone.0356765.s004.docx]

# Response to reviewers

**Manuscript:** Binocular combination in the autonomic nervous system (PONE-D-25-66272)

We thank the editor and both reviewers for their careful and constructive reading. The comments have helped us make the rationale for the experiments, the harmonic analysis, the model, and the physiological interpretation much clearer. Below we respond to each comment in turn. The reviewers' words are quoted in *italics*; our response and the location of each change follow. All data underlying the findings remain available in the reproducible repository.

## Reviewer 1

We are grateful for the nice assessment ("*this paper is neatly done and* *offers valuable data*") and for the clear diagnosis that the paper needed more context. We have worked to supply it.

### Comment 1.1: Rationale for the conditions

*Why include the dichoptic stimulation and the cross-frequency conditions? What* *could we learn, conceptually, from including these conditions? I think the paper* *would be much more readable if the introduction would state in clear and concrete* *terms why all of the manipulations were included.*

We agree. We rewrote the final paragraph of the Introduction to state what each comparison estimates: presenting the two eyes with different temporal frequencies (0.5 and 0.4 Hz) lets us recover each eye's response separately in the Fourier spectrum, comparing monocular and binocular stimulation measures the net effect of adding a second eye but cannot separate extra excitation from suppression and so we use the dichoptic mask to isolate just the suppression one eye exerts on the other.

**Location:** Introduction, final paragraph (and the condition table, Table 2).

### Comment 1.2: What does the second harmonic mean?

*Pupils are not guitar strings. They do not resonate... Simply noting that there* *is a second harmonic, while technically correct, isn't by itself useful. Because:* *What does this reflect and why is it informative to look at it?*

We agree and have added an explanation. A purely linear system driven by a sinusoid responds only at the stimulus frequency, so energy at the second harmonic must reflect a nonlinear transformation of the response waveform (e.g. rectification or asymmetry between constriction and re-dilation) rather than resonance. We now treat the second harmonic as a measure of nonlinear temporal processing, not a separate oscillatory mechanism, and state that this analysis was exploratory.

**Location:** Results (first subsection); Discussion (new physiological-interpretation subsection).

### Comment 1.3: Intelligibility and standard English

*Is the manuscript presented in an intelligible fashion and written in standard* *English?* — Reviewer 1: **No.**

We have improved the writing: we tightened wording, replaced 'Latin' words with plainer ones and generally simplified. The added context above (Comments 1.1 and 1.2) should also make the manuscript easier to follow.

**Location:** throughout.

## Reviewer 2

We thank the reviewer for judging the experiments "*well performed*" and the results "*interesting*", and for the specific concerns about presentation and interpretation, which we address below.

### Comment 2.1: The dichoptic condition uses different frequencies

*The reader was for some time at a loss about the dichoptic stimulation. It would* *have been helpful if it would have been made clear from the beginning that in the* *dichoptic condition the stimulus in the two eyes had different frequencies.*

We now explain the frequency-tagging logic in the Introduction, and we added a table of the six ocular conditions (target eye, other eye, the frequency at which the response is analysed, and the purpose of each), so that the cross-frequency arrangement is clear from the outset.

**Location:** Introduction, final paragraph; Materials and methods, Table 1.

### Comment 2.2: Describe the model in physiological terms

*It might be helpful to describe the model in physiological terms.*

We added a plain-language description before the equations: each eye produces an excitatory response that grows with the contrast in that eye; this is divided by a term that includes the contrast in the other eye, so one eye suppresses the other; the two suppressed signals are summed; and the weight ω sets the strength of interocular suppression (below 1, weaker suppression and stronger facilitation; near or above 1, strong suppression that can give ocularity invariance or a binocular response smaller than the monocular one).

**Location:** Materials and methods (Computational model and parameter estimation).

### Comment 2.3: Were separate fits performed for the two eyes?

*Were separate fits performed for data from left and right eyes?*

No. We now state this explicitly: target-eye assignment was counterbalanced across participants, and responses were pooled across eye before fitting, after confirming that the consensual response matched the response in the stimulated eye. But we note that the model was fitted to the individual-participant amplitudes, not to the group averages.

**Location:** Materials and methods (Computational model and parameter estimation; Data analysis).

### Comment 2.4: Some fits are not convincing (e.g. S-cone 0.4 Hz dichoptic)

*I did not find the fits very convincing in some cases (e.g. the S-cone response* *at 0.4 Hz in dichoptic condition).*

We agree that the fits are not equally good everywhere, and we no longer claim they are. We removed the unqualified "good quality" statement and now say that the model fits the light flux data most closely and the noisier S-cone data least well. We added a table of convergence diagnostics and fit statistics for all eight fits (Table 2: maximum split-R̂, minimum effective sample size, and RMSE) and a posterior predictive check (Figure S3) that plots the model's 95% predictive interval against the data for every condition; the widest intervals and largest deviations are in the noisier, low-amplitude conditions, including the S-cone.

**Location:** Results; Materials and methods (Table 2); Supporting information (Figure S3).

### Comment 2.5: Sum the first and second harmonics for total energy

*I further had the impression that sometimes the 1st harmonic response was very* *low because all energy went into the 2nd harmonic response (e.g., S-cone 96%* *monocular and dichoptic stimulation). Might it be better to sum the responses at* *the 1st and 2nd harmonic to get the total energy and fit the model to this* *addition?*

We agree that energy can shift between the harmonics, so either harmonic alone can misrepresent the total periodic response. Because the harmonics are orthogonal Fourier components, their powers (not their complex amplitudes) add; we therefore computed the root-sum-square of the first- and second-harmonic amplitudes as a combined measure of total periodic response (new Figure S2). This combined measure preserves the main pattern (binocular facilitation for light flux and S-cone stimulation, near-invariance for L-M and melanopsin) while moderating the most extreme ratios. We report this as a supplementary analysis but retain the harmonic-specific model fits, because the two harmonics reflect different components of the waveform and show distinct binocular interactions, and the present model does not describe how nonlinear transduction generates harmonics. For that reason, we did not refit the model to the summed energy.

**Location:** Results; Supporting information (Figure S2).

### Comment 2.6: Discuss the physiology behind the results

*I would have welcomed a discussion about the physiology behind the results. What* *are the differences between the four different pathways and how can they be* *explained? What can explain the substantial differences between the 1st and 2nd* *harmonic responses in the dichoptic condition at 0.4 Hz and 0.5 Hz?*

We added a Discussion subsection, "Physiological interpretation of the pathway and harmonic differences," covering the light flux, L-M, S-cone and melanopsin conditions and the frequency comparison. We are explicit that silent substitution isolates photoreceptor *contrast* rather than an anatomically separate pathway, that the targeted signals converge downstream, and that rods were not explicitly silenced. We interpret the 0.4 versus 0.5 Hz differences cautiously: a temporally filtered nonlinear system can change response phase, waveform asymmetry and harmonic content with a small change in frequency, and some of the difference may reflect noisier estimates or imperfect fit, so we do not try to assign these differences to distinct physiological mechanisms.

**Location:** Discussion (new subsection).

# Overall

We believe the revised manuscript addresses the reviewers' concerns, and we thank them again for comments that have improved the paper.
