# Supplementary material for: Binocular combination in the autonomic nervous system
Source: PLoS One. 2026 Sep 1;21(9):e0356765. doi: 10.1371/journal.pone.0356765 (PMC13533428; doi:10.1371/journal.pone.0356765)
Supplement: Peer review history

# Peer review: Decision Letter 0

**DOI:** 10.1371/journal.pone.0356765.r001

**Authors:** Daniel Joyce

9 May 2026

Dear Dr. Segala,

Thank you for submitting your manuscript to PLOS ONE. After careful consideration, we feel that it has merit but does not fully meet PLOS ONE’s publication criteria as it currently stands. Therefore, we invite you to submit a revised version of the manuscript that addresses the points raised during the review process.

Your submission has been evaluated by two reviewers, who recommend some changes to enhance rationale, clarity, and interpretation of the results. I would be grateful if you would please consider this feedback.

Please submit your revised manuscript by Jun 23 2026 11:59PM. If you will need more time than this to complete your revisions, please reply to this message or contact the journal office at plosone@plos.org. When you're ready to submit your revision, log on to https://www.editorialmanager.com/pone/ and select the 'Submissions Needing Revision' folder to locate your manuscript file.

- A letter that responds to each point raised by the academic editor and reviewer(s). You should upload this letter as a separate file labeled 'Response to Reviewers'.
- A marked-up copy of your manuscript that highlights changes made to the original version. You should upload this as a separate file labeled 'Revised Manuscript with Track Changes'.
- An unmarked version of your revised paper without tracked changes. You should upload this as a separate file labeled 'Manuscript'.

If you would like to make changes to your financial disclosure, please include your updated statement in your cover letter. Guidelines for resubmitting your figure files are available below the reviewer comments at the end of this letter.

If applicable, we recommend that you deposit your laboratory protocols in protocols.io to enhance the reproducibility of your results. Protocols.io assigns your protocol its own identifier (DOI) so that it can be cited independently in the future. For instructions see: https://journals.plos.org/plosone/s/submission-guidelines#loc-laboratory-protocols. Additionally, PLOS ONE offers an option for publishing peer-reviewed Lab Protocol articles, which describe protocols hosted on protocols.io. Read more information on sharing protocols at https://plos.org/protocols?utm_medium=editorial-email&utm_source=authorletters&utm_campaign=protocols.

As the corresponding author, your ORCID iD is verified in the submission system and will appear in the published article. PLOS supports the use of ORCID, and we encourage all coauthors to register for an ORCID iD and use it as well. Please encourage your coauthors to verify their ORCID iD within the submission system before final acceptance, as unverified ORCID iDs will not appear in the published article. *Only*  the individual author can complete the verification step; PLOS staff *cannot*  verify ORCID iDs on behalf of authors.

We look forward to receiving your revised manuscript.

Kind regards,

Daniel Joyce

Academic Editor

PLOS One

**Journal Requirements:**

When submitting your revision, we need you to address these additional requirements.

1. Please ensure that your manuscript meets PLOS ONE's style requirements, including those for file naming. The PLOS ONE style templates can be found at

https://journals.plos.org/plosone/s/file?id=wjVg/PLOSOne_formatting_sample_main_body.pdf and

https://journals.plos.org/plosone/s/file?id=ba62/PLOSOne_formatting_sample_title_authors_affiliations.pdf

2. Please note that PLOS One has specific guidelines on code sharing for submissions in which author-generated code underpins the findings in the manuscript. In these cases, we expect all author-generated code to be made available without restrictions upon publication of the work. Please review our guidelines at https://journals.plos.org/plosone/s/materials-and-software-sharing#loc-sharing-code and ensure that your code is shared in a way that follows best practice and facilitates reproducibility and reuse.

3. Please update your submission to use the PLOS LaTeX template. The template and more information on our requirements for LaTeX submissions can be found at http://journals.plos.org/plosone/s/latex.

4. If the reviewer comments include a recommendation to cite specific previously published works, please review and evaluate these publications to determine whether they are relevant and should be cited. There is no requirement to cite these works unless the editor has indicated otherwise.

**Additional Editor Comments:**

Thank you for your submission. It has been evaluated by two reviewers, who recommend some changes to enhance rationale, clarity, and interpretation of the results. I would be grateful if you would please consider this feedback.

[Note: HTML markup is below. Please do not edit.]

Reviewers' comments:

Reviewer's Responses to Questions

**Comments to the Author**

1. Is the manuscript technically sound, and do the data support the conclusions?

Reviewer #1: Yes

Reviewer #2: Partly

**********

2. Has the statistical analysis been performed appropriately and rigorously?

Reviewer #1: Yes

Reviewer #2: N/A

**********

3. Have the authors made all data underlying the findings in their manuscript fully available?

The PLOS Data policy

Reviewer #1: Yes

Reviewer #2: Yes

**********

4. Is the manuscript presented in an intelligible fashion and written in standard English?

Reviewer #1: No

Reviewer #2: Yes

**********

Reviewer #1: # Review

Segala et al. (2026) Binocular combination in the autonomic nervous system. *PLoS ONE*.

---

In this article, the authors record pupil responses of a target eye to 0.5 Hz flickering stimuli that stimulate either L-M cones, S-cones, or melanopsin/ ipRGCs. They also vary whether stimuli are presented monocularly (to the target eye), binocularly, or accompanied by a dichoptic mask consisting of a different flickering stimulus presented to the non-target eye. They also include a cross-frequency condition in which they do the same thing, except that the flicker in the non-target eye is 0.4 Hz (i.e. different from the target frequency).

My background is in cognitive neuroscience, and I'm not very familiar with the exact methodology. But to the extent that I can judge, the authors conducted their experiments appropriately.

To me, the main point of improvement is context. Right now, the manuscript is difficult to parse, because very little reason is provided for why the authors did the experiments that they did, or what their results mean. For example, I understand why you'd compare monocular and binocular presentation—so far so good. But why include the dichoptic stimulation and the cross-frequency conditions? What could we learn, conceptually, from including these conditions? I think the paper would be much more readable if the introduction would state in clear and concrete terms why all of the manipulations were included.

I felt similarly about the results, and especially the focus on the second harmonic. Of course I can see in the figures that there is indeed something that resembles a 1 Hz oscillation. But what does this mean? Pupils are not guitar strings. They do not resonate. Rather, the pupil light response is a more or less stereotyped constriction followed (in case of brief stimuli) by a re-dilation. Depending on stimulation frequency and intensity, the pupil timeseries, which of course results from many partly overlapping pupil responses, can take on many forms. Simply noting that there is a second harmonic, while technically correct, isn't by itself useful. Because: What does this reflect and why is it informative to look at it?

In sum, I think this paper is neatly done and offers valuable data. But it would be much stronger if the authors provide much more context to help the reader understand why they conducted this study, and what their results mean.

---

Sebastiaan Mathôt \

Department of Experiment Psychology \

University of Groningen \

<http: smathot=“” www.cogsci.nl=“”></http:>

Reviewer #2: Review of the manuscript “Binocular combination in the autonomic nervous system” (PONE-D-25-66272) by Segala et al.

In this manuscript the results of pupillary response measurements are described, while stimulating either monocularly, binocularly or dichoptically. Stimuli were either luminance, S-cone directed, melanopsin directed or L-M opponent, thereby stimulating different retinal pathways. The results showed that different pathways may also have different effects on the pupillary response and the interocular interaction may be different.

The experiments were well performed and the results are interesting. I, however, had problems with the presentation of the results and the interpretation of the data.

The reader was for some time at a loss about the dichoptic stimulation. It would have been helpful if it would have been made clear from the beginning that in the dichoptic condition the stimulus in the two eyes had different frequencies.

The data were fitted by a model, that is claimed to describe the data relatively well. It might be helpful to describe the model in physiological terms. Were separate fits performed for data from left and right eyes? If yes, then I did not find the fits very convincing in some cases (e.g. the S-cone response at 0.4 Hz in dichoptic condition).

I further had the impression that sometimes the 1st harmonic response was very low because all energy went into the 2nd harmonic response (e.g., S-cone 96% monocular and dichoptic stimulation) might it be better to sum the responses at the 1st and 2nd harmonic to get the total energy and fit the model to this addition?

Furthermore, I would have welcomed a discussion about the physiology behind the results. What are the differences between the four different pathways and how can they be explained? What can explain the substantial differences between the 1st and 2nd harmonic responses in the dichoptic condition at 0.4 Hz and 0. 5 Hz?

**********

what does this mean?). If published, this will include your full peer review and any attached files.

If you choose “no”, your identity will remain anonymous but your review may still be made public.

**Do you want your identity to be public for this peer review?**  For information about this choice, including consent withdrawal, please see our Privacy Policy

Reviewer #1: **Yes:**  Sebastiaan Mathôt

Reviewer #2: No

**********

[NOTE: If reviewer comments were submitted as an attachment file, they will be attached to this email and accessible via the submission site. Please log into your account, locate the manuscript record, and check for the action link "View Attachments". If this link does not appear, there are no attachment files.]

To ensure your figures meet our technical requirements, please review our figure guidelines: https://journals.plos.org/plosone/s/figures

You may also use PLOS’s free figure tool, NAAS, to help you prepare publication quality figures: https://journals.plos.org/plosone/s/figures#loc-tools-for-figure-preparation.

NAAS will assess whether your figures meet our technical requirements by comparing each figure against our figure specifications.

# Author comment: Author response to Decision Letter 1

**DOI:** 10.1371/journal.pone.0356765.r002

17 Jul 2026

Response to reviewers

Manuscript: Binocular combination in the autonomic nervous system (PONE-D-25-66272)

We thank the editor and both reviewers for their careful and constructive reading. The comments have helped us make the rationale for the experiments, the harmonic analysis, the model, and the physiological interpretation much clearer. Below we respond to each comment in turn. The reviewers' words are quoted in italics; our response and the location of each change follow. All data underlying the findings remain available in the reproducible repository.

Reviewer 1

We are grateful for the nice assessment ("this paper is neatly done and offers valuable data") and for the clear diagnosis that the paper needed more context. We have worked to supply it.

Comment 1.1: Rationale for the conditions

Why include the dichoptic stimulation and the cross-frequency conditions? What could we learn, conceptually, from including these conditions? I think the paper would be much more readable if the introduction would state in clear and concrete terms why all of the manipulations were included.

We agree. We rewrote the final paragraph of the Introduction to state what each comparison estimates: presenting the two eyes with different temporal frequencies (0.5 and 0.4 Hz) lets us recover each eye's response separately in the Fourier spectrum, comparing monocular and binocular stimulation measures the net effect of adding a second eye but cannot separate extra excitation from suppression and so we use the dichoptic mask to isolate just the suppression one eye exerts on the other.

Location: Introduction, final paragraph (and the condition table, Table 2).

Comment 1.2: What does the second harmonic mean?

Pupils are not guitar strings. They do not resonate... Simply noting that there is a second harmonic, while technically correct, isn't by itself useful. Because: What does this reflect and why is it informative to look at it?

We agree and have added an explanation. A purely linear system driven by a sinusoid responds only at the stimulus frequency, so energy at the second harmonic must reflect a nonlinear transformation of the response waveform (e.g. rectification or asymmetry between constriction and re-dilation) rather than resonance. We now treat the second harmonic as a measure of nonlinear temporal processing, not a separate oscillatory mechanism, and state that this analysis was exploratory.

Location: Results (first subsection); Discussion (new physiological-interpretation subsection).

Comment 1.3: Intelligibility and standard English

Is the manuscript presented in an intelligible fashion and written in standard English? — Reviewer 1: No.

We have improved the writing: we tightened wording, replaced 'Latin' words with plainer ones and generally simplified. The added context above (Comments 1.1 and 1.2) should also make the manuscript easier to follow.

Location: throughout.

Reviewer 2

We thank the reviewer for judging the experiments "well performed" and the results "interesting", and for the specific concerns about presentation and interpretation, which we address below.

Comment 2.1: The dichoptic condition uses different frequencies

The reader was for some time at a loss about the dichoptic stimulation. It would have been helpful if it would have been made clear from the beginning that in the dichoptic condition the stimulus in the two eyes had different frequencies.

We now explain the frequency-tagging logic in the Introduction, and we added a table of the six ocular conditions (target eye, other eye, the frequency at which the response is analysed, and the purpose of each), so that the cross-frequency arrangement is clear from the outset.

Location: Introduction, final paragraph; Materials and methods, Table 1.

Comment 2.2: Describe the model in physiological terms

It might be helpful to describe the model in physiological terms.

We added a plain-language description before the equations: each eye produces an excitatory response that grows with the contrast in that eye; this is divided by a term that includes the contrast in the other eye, so one eye suppresses the other; the two suppressed signals are summed; and the weight ω sets the strength of interocular suppression (below 1, weaker suppression and stronger facilitation; near or above 1, strong suppression that can give ocularity invariance or a binocular response smaller than the monocular one).

Location: Materials and methods (Computational model and parameter estimation).

Comment 2.3: Were separate fits performed for the two eyes? Were separate fits performed for data from left and right eyes?

No. We now state this explicitly: target-eye assignment was counterbalanced across participants, and responses were pooled across eye before fitting, after confirming that the consensual response matched the response in the stimulated eye. But we note that the model was fitted to the individual-participant amplitudes, not to the group averages.

Location: Materials and methods (Computational model and parameter estimation; Data analysis).

Comment 2.4: Some fits are not convincing (e.g. S-cone 0.4 Hz dichoptic)

I did not find the fits very convincing in some cases (e.g. the S-cone response at 0.4 Hz in dichoptic condition).

We agree that the fits are not equally good everywhere, and we no longer claim they are. We removed the unqualified "good quality" statement and now say that the model fits the light flux data most closely and the noisier S-cone data least well. We added a table of convergence diagnostics and fit statistics for all eight fits (Table 2: maximum split-R̂, minimum effective sample size, and RMSE) and a posterior predictive check (Figure S3) that plots the model's 95% predictive interval against the data for every condition; the widest intervals and largest deviations are in the noisier, low-amplitude conditions, including the S-cone.

Location: Results; Materials and methods (Table 2); Supporting information (Figure S3).

Comment 2.5: Sum the first and second harmonics for total energy

I further had the impression that sometimes the 1st harmonic response was very low because all energy went into the 2nd harmonic response (e.g., S-cone 96% monocular and dichoptic stimulation). Might it be better to sum the responses at the 1st and 2nd harmonic to get the total energy and fit the model to this addition?

We agree that energy can shift between the harmonics, so either harmonic alone can misrepresent the total periodic response. Because the harmonics are orthogonal Fourier components, their powers (not their complex amplitudes) add; we therefore computed the root-sum-square of the first- and second-harmonic amplitudes as a combined measure of total periodic response (new Figure S2). This combined measure preserves the main pattern (binocular facilitation for light flux and S-cone stimulation, near-invariance for L-M and melanopsin) while moderating the most extreme ratios. We report this as a supplementary analysis but retain the harmonic-specific model fits, because the two harmonics reflect different components of the waveform and show distinct binocular interactions, and the present model does not describe how nonlinear transduction generates harmonics. For that reason, we did not refit the model to the summed energy.

Location: Results; Supporting information (Figure S2).

Comment 2.6: Discuss the physiology behind the results

I would have welcomed a discussion about the physiology behind the results. What are the differences between the four different pathways and how can they be explained? What can explain the substantial differences between the 1st and 2nd harmonic responses in the dichoptic condition at 0.4 Hz and 0.5 Hz?

We added a Discussion subsection, "Physiological interpretation of the pathway and harmonic differences," covering the light flux, L-M, S-cone and melanopsin conditions and the frequency comparison. We are explicit that silent substitution isolates photoreceptor contrast rather than an anatomically separate pathway, that the targeted signals converge downstream, and that rods were not explicitly silenced. We interpret the 0.4 versus 0.5 Hz differences cautiously: a temporally filtered nonlinear system can change response phase, waveform asymmetry and harmonic content with a small change in frequency, and some of the difference may reflect noisier estimates or imperfect fit, so we do not try to assign these differences to distinct physiological mechanisms.

Location: Discussion (new subsection).

Overall, we believe the revised manuscript addresses the reviewers' concerns, and we thank them again for comments that have improved the paper.

Attachment Submitted filename: response_to_reviewers.docx

# Peer review: Decision Letter 1

**DOI:** 10.1371/journal.pone.0356765.r003

**Authors:** Daniel Joyce

10 Aug 2026

Binocular combination in the autonomic nervous system

PONE-D-25-66272R1

Dear Dr. Segala,

We’re pleased to inform you that your manuscript has been judged scientifically suitable for publication and will be formally accepted for publication once it meets all outstanding technical requirements.

Within one week, you’ll receive an e-mail detailing the required amendments. When these have been addressed, you’ll receive a formal acceptance letter and your manuscript will be scheduled for publication.

An invoice will be generated when your article is formally accepted. Please note, if your institution has a publishing partnership with PLOS and your article meets the relevant criteria, all or part of your publication costs will be covered. Please make sure your user information is up-to-date by logging into Editorial Manager at Editorial Manager® and clicking the ‘Update My Information' link at the top of the page. For questions related to billing, please contact billing support.

If your institution or institutions have a press office, please notify them about your upcoming paper to help maximize its impact. If they’ll be preparing press materials, please inform our press team as soon as possible -- no later than 48 hours after receiving the formal acceptance. Your manuscript will remain under strict press embargo until 2 pm Eastern Time on the date of publication. For more information, please contact onepress@plos.org.

Kind regards,

Daniel Joyce

Academic Editor

PLOS One

Additional Editor Comments (optional):

Thank you for considering the comments from both reviwers. The manuscript is much improved.

Reviewers' comments:

Reviewer's Responses to Questions

**Comments to the Author**

Reviewer #1: All comments have been addressed

Reviewer #2: All comments have been addressed

**********

2. Is the manuscript technically sound, and do the data support the conclusions?

Reviewer #1: Yes

Reviewer #2: (No Response)

**********

3. Has the statistical analysis been performed appropriately and rigorously?

Reviewer #1: Yes

Reviewer #2: Yes

**********

4. Have the authors made all data underlying the findings in their manuscript fully available?

The PLOS Data policy

Reviewer #1: Yes

Reviewer #2: (No Response)

**********

5. Is the manuscript presented in an intelligible fashion and written in standard English?

Reviewer #1: Yes

Reviewer #2: (No Response)

**********

Reviewer #1: (No Response)

Reviewer #2: The authors have addressed the suggestions and the manuscript has improved. I congratulate the authors with a fine paper.

**********

what does this mean?). If published, this will include your full peer review and any attached files.

If you choose “no”, your identity will remain anonymous but your review may still be made public.

**Do you want your identity to be public for this peer review?**  For information about this choice, including consent withdrawal, please see our Privacy Policy

Reviewer #1: **Yes:**  Sebastiaan Mathôt

Reviewer #2: **Yes:**  Jan Kremers

**********

# Editor report: Acceptance letter

**DOI:** 10.1371/journal.pone.0356765.r004

**Authors:** Daniel Joyce

PONE-D-25-66272R1

PLOS One

Dear Dr. Segala,

I'm pleased to inform you that your manuscript has been deemed suitable for publication in PLOS One. Congratulations! Your manuscript is now being handed over to our production team.

At this stage, our production department will prepare your paper for publication. This includes ensuring the following:

* All references, tables, and figures are properly cited

* All relevant supporting information is included in the manuscript submission,

* There are no issues that prevent the paper from being properly typeset

You will receive further instructions from the production team, including instructions on how to review your proof when it is ready. Please keep in mind that we are working through a large volume of accepted articles, so please give us a few days to review your paper and let you know the next and final steps.

Lastly, if your institution or institutions have a press office, please let them know about your upcoming paper now to help maximize its impact. If they'll be preparing press materials, please inform our press team within the next 48 hours. Your manuscript will remain under strict press embargo until 2 pm Eastern Time on the date of publication. For more information, please contact onepress@plos.org.

You will receive an invoice from PLOS for your publication fee after your manuscript has reached the completed accept phase. If you receive an email requesting payment before acceptance or for any other service, this may be a phishing scheme. Learn how to identify phishing emails and protect your accounts at https://explore.plos.org/phishing.

If we can help with anything else, please email us at customercare@plos.org.

Thank you for submitting your work to PLOS One and supporting open access.

Kind regards,

PLOS ONE Editorial Office Staff

on behalf of

Dr. Daniel Joyce

Academic Editor

PLOS One
